# Analysis of *TMIE* gene mutations including the first large deletion of exon 1 with autosomal recessive non-syndromic deafness

**DOI:** 10.1186/s12920-022-01287-9

**Published:** 2022-06-16

**Authors:** Sima Rayat, Mohammad Farhadi, Hessamaldin Emamdjomeh, Saeid Morovvati, Masoumeh Falah

**Affiliations:** 1 ENT and Head and Neck Research Center, The Five Senses Health Institute, School of Medicine, Iran University of Medical Sciences, Tehran, Iran; 2 Department of Biology, School of Basic Sciences, Science and Research Branch, Islamic Azad University, Tehran, Iran; 3 Department of Genetics, Faculty of Advanced Sciences and Technology, Tehran Medical Sciences, Islamic Azad University, Tehran, Iran

**Keywords:** Hearing loss, DFNB6, *TMIE*, Whole-exome sequencing, CNV

## Abstract

**Background:**

Transmembrane inner ear (TMIE) protein is an essential component of the mechanotransduction complex. In collaboration with other components, TMIE aids the maintenance and function of the sensory hair cells. Autosomal recessive deafness-6 (DFNB6) is caused by mutated *TMIE*, a gene in the high genetic heterogeneity spectrum of deafness. Hearing loss has a significant impact on the global economy and the quality of life of affected persons, their families, and society. Here, three unrelated families with *TMIE* variants are presented. All three cases were found while studying the genetic causes of an Iranian cohort of subjects with cochlear implants.

**Methods:**

Whole exome sequencing was performed to find possible genetic etiology in probands of families after a comprehensive medical evaluation for hearing loss. Co-segregation analysis in probands and other family members was performed by Sanger sequencing. The variants were interpreted per the American College of Medical Genetics and Genomics guidelines.

**Results:**

Three different variants associated with *TMIE* were confirmed as reasons for autosomal recessive non-syndromic deafness. The first novel ~ 10-kb deletion surrounding exon 1 of *TMIE* along with two previously reported variants co-segregated with families including a frameshift variant c.122_125dup (p.Pro43fs) and a missense variant c.250 C > T; p.(Arg84Trp) in exons 2, and 3, respectively.

**Conclusion:**

This study increases the mutational spectrum of the *TMIE* gene and highlights the importance of the large deletion of this gene as a reason for hearing loss. Moreover, an efficient and simple multiplex PCR assay was developed to determine the exact breakpoints of the *TMIE* deletion.

**Supplementary Information:**

The online version contains supplementary material available at 10.1186/s12920-022-01287-9.

## Introduction

Hearing loss (HL) is the most prevalent sensory impairment, affecting one in every 500 newborns [1, 2]. Currently, HL affects more than 1.5 billion of the world’s population, about one in five people, and is expected to affect more than 2·45 billion people in 2050 [3, 4]. HL significantly impacts the global economy and quality of life of affected persons, their families, and society [5–7].

HL is a highly heterogeneous disorder that results from a wide range of environmental factors, including ototoxic drugs, infections, neonatal complications, and more than 150 auditory development-related genes [8–11]. In fact, genes are responsible for more than 60% of prelingual HL [12]. HL can be classified into different categories based on various factors, including type (conductive, sensorineural, mixed, and central auditory dysfunction), onset (prelingual and postlingual), severity (mild, moderate, moderately severe, severe, and profound), and whether it is isolated (non-syndromic) or associated with other clinical issues (syndromic) [13].

In the last two decades, new generations of sequencing have been applied, and a wide range of HL-related mutations have been uncovered [14]. These factors have provided valuable information about the genotype–phenotype correlation to improve the effectiveness of genetic counseling and prognoses for the disease [2, 15]. Eighty percent of hearing impairment are caused by genes with autosomal recessive inheritance; autosomal dominant, X-linked, and mitochondrial inheritance are less common. The diagnostic rate of hearing impairment-related genes depends heavily on family history, phenotype, onset time, patient ethnicity, and symmetry in HL [2].

To date, 78 genes are known to cause autosomal recessive non-syndromic HL (https://hereditaryhearingloss.org). The transmembrane inner ear (*TMIE*) gene participates in the mechanotransduction machinery of hair cells. Loss-of-function mutations in the evolutionarily conserved protein TMIE were causally associated with autosomal recessive deafness-6 (DFNB6; OMIM: 600,971) in human and animal studies [16–19]. DFNB6 is categorized as a type of severe-to-profound congenital or prelingual HL [16]. *TMIE* (OMIM: 607,237), with four exons, is located on the 3p21 chromosomal region. *TMIE* produces a protein with two transmembrane domains joined by an extracellular loop, plus N and C intracellular terminus, which play a role in the hearing process by aiding the maintenance, maturation, and development of the inner ear’s sensory hair cells [17, 20, 21].

In the present investigation of the genetic background of an Iranian cohort with cochlear implants, we describe three unrelated families with autosomal recessive non-syndromic HL who exhibited mutations in the *TMIE* gene using whole-exome sequencing (WES). Moreover, we introduced a reliable and simple PCR method for finding the boundaries of the novel deletion of the *TMIE* gene.

## Materials and methods

### Ethics statement

This study was approved by the Ethics Committee of the Iran University of Medical Sciences (Tehran, Iran) according to national law and the World Medical Association Code of Ethical Principles for Medical Research Involving Human Subjects. (Approval number: IR.IUMS.REC.1400.862). Written consent was obtained from all participants according to the Declaration of Helsinki.

### Study participants and clinical evaluations

**Fig. 1:**
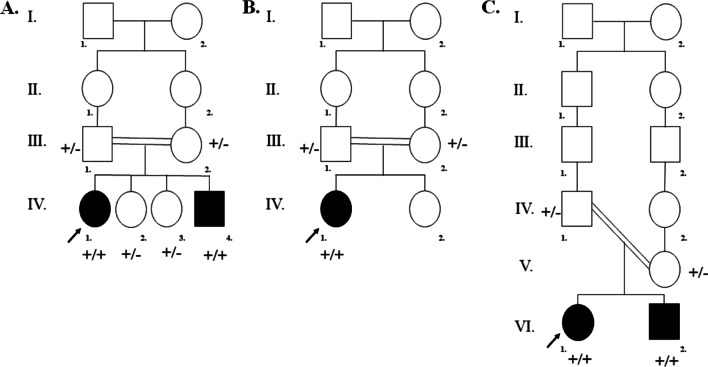
Pedigree information showing segregation of *TMIE* variants. **A** Pedigree of family 1. ( +): a ~ 10-kb deletion **B** Pedigree of family 2. ( +): c.122_125dup (p.Pro43fs) **C** Pedigree of family 3. ( +): c. 250 C > T; p.(Arg84Trp). The ( −) indicates the wild-type allele. The arrows show the affected individual who was selected for whole-exome sequencing. In these figures, white symbols signify unaffected; black symbols mean affected; squares are men; circles are females; parallel lines show consanguineous marriage

Three consanguineous, unrelated Iranian families with siblings affected with HL were recruited (Fig. 1). The proband of the first family (IV.I) was a 17-year-old female; her 8-year-old brother also had prelingual HL (Fig. 1A). The proband of the second family (IV.I) was a 6-year-old female (Fig. 1B). and the proband of the third family (VI.I) was a 17-year-old female. The third family had only two children, both of whom had prelingual HL (Fig. 1C). The consanguineous parents and healthy siblings of families had normal auditory and verbal functions. Clinical evaluations, including family history and physical examinations for hearing, as well as tests for the presence of associated symptoms in syndromes and exposure to environmental factors, were performed in Hazrat Rasoul Akram Hospital, Tehran, Iran [22].

Individuals IV.1, IV.4 (family 1), IV.1 (family 2), and IV.1, VI.1, VI.2 (family3) (Fig. 1) underwent complete ear, nose and throat examination. Routine pure tone audiometry was performed on IV.1 from family 3 according to current standards. Air and bone conduction thresholds were measured at 250–8000 and 250–4000 Hz, respectively [23]. Audiological examinations, including auditory brainstem response, pure tone otoacoustic emission, and auditory steady-state response, were carried out to determine hearing thresholds for individuals IV.1, IV.4 (family 1), IV.1 (family 2), and VI.1, VI.2 (family3).

Blood samples (5 mL) were collected from the contributors, and genomic DNA was extracted as previously described by Falah et al. [9].

### Whole-exome sequencing

WES was performed based on previous work [15]. In short, WES enrichment was performed on the probands’ genomic DNA using a Twist Human Core Exome Kit (Twist Bioscience, CA, USA) on an Illumina NovaSeq system (Illumina, San Diego, CA, USA) provided by CeGaT GmbH Company (Tübingen, Germany). The reads were aligned to the GRCh38/hg38 human reference genome sequence. The analytical sensitivity and specificity for detecting point mutations, micro-insertion, deletion, and duplication (within 20 bp) was > 95%.

The stepwise data analysis method was performed to find the causative variants. The first step was to remove variants with a minor allele frequency of > 1% in public databases such as gnomAD [24], 1000 Genome [25], dbSNP [26], and Iranome [27]. In the second step, all non-coding regions other than the 20 bp flanking regions and synonymous variants were excluded. The results of variants were predicted using bioinformatics tools, including MutationTaster [28], Polyphen2 [29], SIFT [30], Provean [31], and Combined Annotation Dependent Depletion [32]. Human Splicing Finder v.3.1 was applied to find the role of variants in the splicing process [33]. The Berkeley Drosophila Genome Project and NetGene2 were used to analyze the effects of the variant on mRNA splicing [34, 35]. The remaining variants were ranked in order of priority according to patient phenotypes. (e.g., hearing symptoms) using Human Gene Mutation Database (HGMD) [36], ClinVar [37], Deafness Variation Database (DVD) [8], and human phenotype ontology [38]. ACMG/AMP (American College of Genetics and Genomics/Association for Molecular Pathology) guidelines were used for variant interpretation in the context of HL [39].

### Variant validation

The identified variants were verified by direct Sanger sequencing in patients, and co-segregation analysis of candidate variants was performed using the samples obtained from parents and selected healthy relatives. Primers for the region of the candidate variants were designed using Primer3 (Table 1) [40]. PCRs were performed in a total volume of 25 μl, including 12 μl 2 × Taq DNA Polymerase Master Mix RED (Ampliqon, Odense, Denmark), 1 μl of each primer described in (Table 1), 2 μl of genomic DNA, and 9 μl of ddH2O. PCRs were performed with the initial denaturation at 95 °C for 4.0 min, followed by 30 cycles at 95 °C for 45 s, at 58 °C for 45 s, at 72 °C for 45 s, and a final extension at 72 °C for 7.0 min.

**Table 1:** Primer sequences and products size of the *TMIE* gene

Family	Exon number	Primer name	Primer sequence (5′–3′)	Product size ( bp)
1	Upstream Exon 1	F1	5′- AGAATCGAATTGGAAGGCAC-3′	184
		R1	5′-TTAAGGCGAACATCCTGAAAG-3′	
	Upstream Exon 1	F2	5′-CTGTGAGCGGGGTATCTTAC-3′	326
		R2	5′-CGCAACTCCAGAGCAAC-3′	
	Downstream Exon 1	F3	5′-CAGCCCACAGATCCTCTG-3′	225
		R3	5′-TAAATAAGGACAGACACGAGAAGTC-3′	
	Multiplex Exon 1	F1	5′-AGAATCGAATTGGAAGGCAC-3′	184
		R1	5′-TTAAGGCGAACATCCTGAAAG-3′	
		F2	5′-CTGTGAGCGGGGTATCTTAC-3′	
		R3	5′-TAAATAAGGACAGACACGAGAAGTC-3′	1111
2	Exon 2	F4	5́-CTCCCACTTCAAGTACCTGGCTC-3́	616
		R4	5́-CACGTCCTCGTCCCAGTCC-3́	
3	Exon 3	F5	5́-CCATTCCTTGGGTCTCTGAA-3́	288
		R5	5́-AGCAGAGGAACAGGGTGAC-3́	

In family 1, the primers (Table 1) were used to find the exact breakpoints of the *TMIE* deletion according to the following method. In the first stage, PCRs were performed with two pairs of upstream primers 1 and 2 and one pair of downstream primer 3 of the deleted exon of the *TMIE* gene. In the next step, in multiplex PCR, all four primers (F2, R3, and the pair of primers 1) were used in a single reaction tube. The PCR products were visualized using ethidium bromide staining and 2% agarose gel electrophoresis. The purified PCR product of affected IV.1 was directly sequenced in forward directions using a PRISM 3100 sequencer (Applied Biosystems, Foster City, CA, USA). Sequencing results were analyzed by Codon code aligner software version 6.0.2 (CodonCode Corp).

In families 2 and 3, the PCR products with primers (F4, R4) and (F5, R5) were used for direct Sanger sequencing (Table 1).

## Results

### Clinical presentation

Clinical examinations and audiology evaluations showed that the three unrelated families (Fig. 1; Table 2) had non-syndromic severe-to-profound HL with an autosomal recessive inheritance pattern. Imaging investigations and computed tomography scans did not reveal any abnormalities in the anatomical structures of the middle and inner parts of the patients’ ears. The cochlear implant surgery was previously performed for all affected persons (Table 2). After cochlear implantation, the auditory-verbal therapy was provided twice every week for 1 year. The result of categories of auditory performance-II (CAP-II) and speech intelligibility rating (SIR) scores were evaluated after the first year of implantation (Table 2, Additional file 1) [41, 42].

**Table 2:** Summary of genotype and clinical characteristics of three unrelated Iranian families

Family	Pedigree	Variation					Age at test	Age of onset	Age at cochlear implant	Type of HL	CAP-II	SIR	Gender
		Chromosome	Nucleotide	Protein	Type	Status							
Family 1	IV.I	Chr3:46,694,176–46,703,459	g.46694176-46703459del	–	Deletion	Hom	17y	Prelingual	5y	Profound	7/9	4/5	Female
	IV.4	Chr3:46,694,176–46,703,459	g.46694176-46703459del	–	Deletion	Hom	8y	Prelingual	5y	Profound	6/9	3/5	Male
	IV.2	Chr3:46,694,176–46,703,459	g.46694176-46703459del	–	Deletion	Het	12y	–	–	Normal	–	–	Female
	IV.3	Chr3:46,694,176–46,703,459	g.46694176-46703459del	–	Deletion	Het	11y	–	–	Normal	–	–	Female
	III.1	Chr3:46,694,176–46,703,459	g.46694176-46703459del	–	Deletion	Het	40y	–	–	Normal	–	–	Male
	III.2	Chr3:46,694,176–46,703,459	g.46694176-46703459del	–	Deletion	Het	36y	–	–	Normal	–	–	Female
Family 2	IV.I	Chr3: 46,705,816–46,705,817	c.122_125dup	p.Pro43AlafsTer73	Frameshift	Hom	6y	Prelingual	10 m	Severe to profound	7/9	5/5	Female
	III.1	Chr3: 46,705,816–46,705,817	c.122_125dup	p.Pro43AlafsTer73	Frameshift	Het	37y	–	–	Normal	–	–	Male
	III.2	Chr3: 46,705,816–46,705,817	c.122_125dup	p.Pro43AlafsTer73	Frameshift	Het	30y	–	–	Normal	–	–	Female
Family 3	VI.1	Chr3: 46,709,164	c.250 C > T	p.(Arg84Trp)	Missense	Hom	17y	Prelingual	10y	Profound	6/9	4/5	Female
	VI.2	Chr3: 46,709,164	c.250 C > T	p.(Arg84Trp)	Missense	Hom	7y	Prelingual	10 m	Profound	7/9	5/5	Male
	V.1	Chr3: 46,709,164	c.250 C > T	p.(Arg84Trp)	Missense	Het	38y	–	–	Normal	–	–	Female
	IV.1	Chr3: 46,709,164	c.250 C > T	p.(Arg84Trp)	Missense	Het	36y	–	–	Normal	–	–	Male

The annotation was applied according to the Homo sapiens genome assembly GRCh38 (hg 38)

*Hom* homozygote, *Het* heterozygote, *Y* year, *M* month, *CAP II* categories of auditory performance, *SIR* speech intelligibility rating

### Molecular findings

In family 1, the WES results indicated a ~ 10-kb deletion region in proband IV.1, spanning across exon 1 of the *TMIE* gene (NM_147196.3), likely causing abnormal gene translation or nonsense-mediated mRNA decay. The deletion was not reported in the DGV [43], gnomAD, ClinVar, and UCSC [44]. The allele frequency was also not reported in the local database, Iranome (in same-ethnically individuals).

**Fig. 2:**
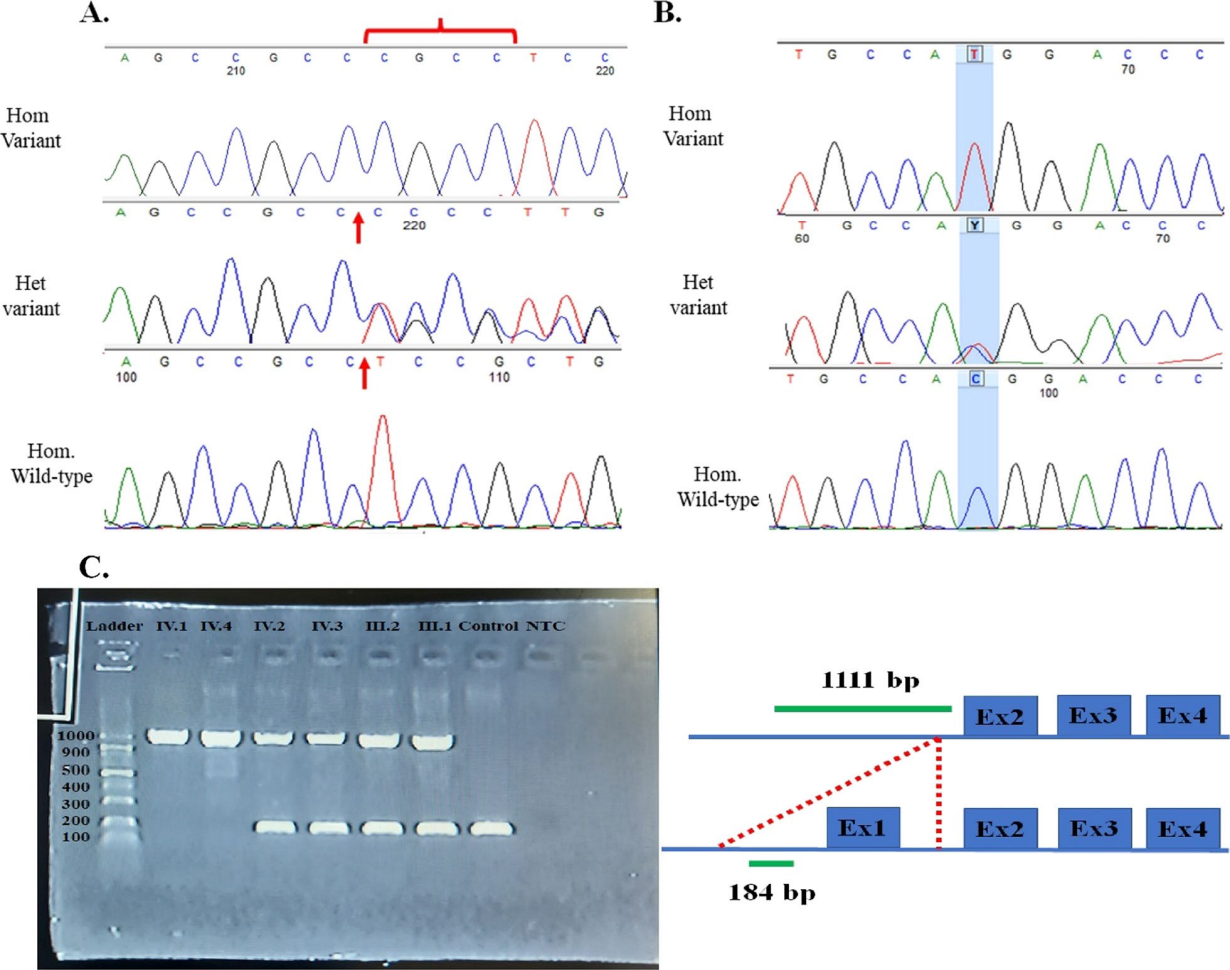
Chromatograms indicate nucleotide sequences of *TMIE*. **A** The c.122_125dup which is found in family 2 in exon 2 of *TMIE*. Duplicated nucleotides are indicated by red arrows and bracket **B** A missense substitution of the c.250C > T variant in exon 3 of *TMIE* is highlighted in blue. Affected probands are homozygous (Hom. for Variant), their parents are heterozygous (Het. for variant), and a normal control subject is homozygous (Hom. for Wild-type allele). **C** Electrophoretogram of the Multiplex-PCR products of a novel ~ 10-Kb deletion in family 1 is indicated on the left side. Lane 1: molecular weight markers, lanes 2 and 3: the homozygous deletions in probands IV.1 and IV.4, respectively, lanes 4–7: the heterozygous deletion in two normal sister, mother and father, respectively, lane 8: the homozygous for wild-type allele in a normal control subject, lane 9: NTC (No Template Control). A schematic depiction of the Multiplex-PCR assay for deletion genotyping is indicated on the right side. A 1111 bp PCR product is observed in subjects that have a ~ 10-kb allele deletion in exon one and its surrounding area of *TMIE*. A 184 bp PCR product is seen in subjects that have a Wild-type allele of *TMIE*

A frameshift variant in the *TMIE* gene—NM_147196.3: c.122_125dup (p.Pro43fs)—was identified that co-segregated with the phenotype in family 2 (Fig. 2A; Table 2). This variant was reported as pathogenic in ClinVar (VCV000984396.1), HGMD, and DVD databases. The variant was classified as “pathogenic” according to ACMG/AMP guidelines (Criteria: PVS1, PM2, and PP5) [39]. Furthermore, a missense variant in the *TMIE* gene—NM_147196.3: c.250 C > T; p.(Arg84Trp)—was identified that co-segregated with the phenotype in family 3 (Fig. 2B; Table 2). This variant was reported as pathogenic/likely pathogenic in ClinVar (VCV000003391.15), HGMD, and DVD databases. The variant was classified as “likely pathogenic” according to ACMG/AMP guidelines (Criteria: PM1, PM2, PM5, PP3, and PP5) [39].

### Validation of a large deletion in *TMIE*

**Fig. 3:**
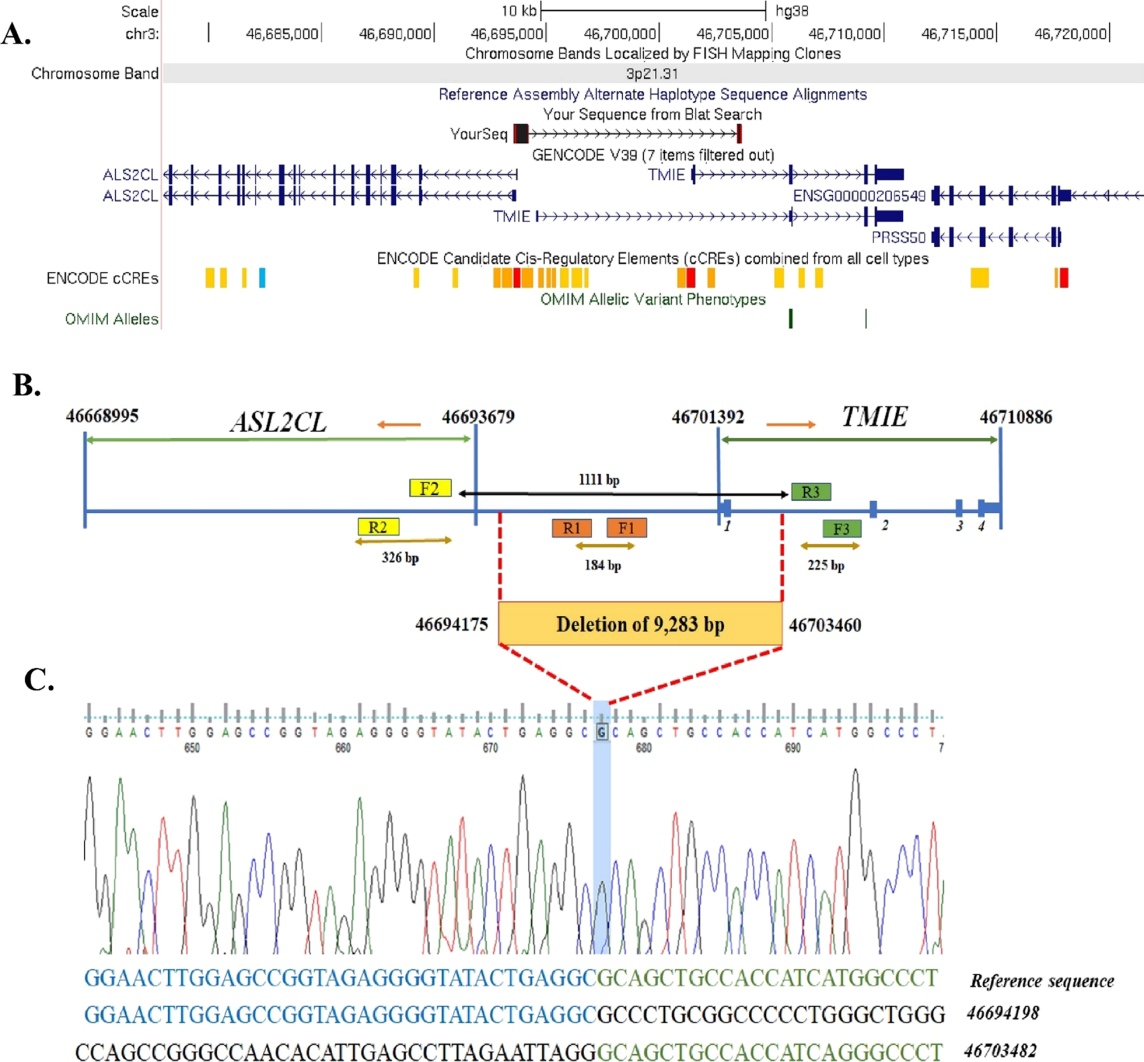
**A** The purified PCR product of the affected individual (IV.1) are indicated in the Blat DNA sequence alignment tool from the UCSC Genome Browser (your seq in the picture). The deleted region covers the exon one of the *TMIE* gene and the surrounding areas. ENCODE Candidate Cis-Regulatory Elements around the deletion part are indicated by red (promoter-like signature), orange (proximal enhancer-like signature), and yellow (distal enhancer-like signature) color. **B** A schematic depiction of the novel 9283 bp deletion and the positions of pair primers 1 and 2 upstream and 3 downstream were used for validation of this deletion in the *TMIE* gene. The nucleotides before and after the deleted region are shown in the image with the numbers 46,694,175 and 46,703,460, respectively. **C** Sanger sequencing electropherograms of the *TMIE* deletion mutation were identified in this study

The product of the first PCR using a pair of F1 and R1 primers upstream of exon 1 had a length of 184 bp. This product was observed in all family members but not in patients, which indicated a lack of this area in patients (Figs. 2C and 3B). The second PCR product had a length of 326 bp and the primer pairs F2 and R2. This product was observed in the upstream area of exon 1 in all family members (Fig. 3B). The third PCR product had a length of 225 bp with primers F3 and R3; this product was also observed in all family members (Fig. 3B), thus indicating the presence of these upstream and downstream parts in all individuals. Finally, a multiplex PCR was performed to find the exact deletion point following an easy, accurate, and cost-effective method using primers F1, R1, F2, and R3. This process led to two products with 184 and 1111 bp. The PCR electrophoretogram of the deleted region is shown in (Fig. 2C). Affected family members (IV.1 and IV.4) showed a 1111-bp band, indicating a ~ 10-kb homozygous deletion. A normal individual without deletion in this region indicated a band with a size of 184 bp. Heterozygous individuals for deletion were seen with two bands (1111 bp and 184 bp) (Figs. 2C and 3B). Direct Sanger sequencing of purified PCR products of an affected individual (IV.1) indicated a novel homozygous deletion of 9283 bp in NC_000003.12: g. 46,694,176-46703459del (Fig. 3). This was classified as a “pathogenic” variant according to the ACMG/AMP guidelines for variant interpretation regarding HL (Criteria: PVS1, PM1, and PM2) [39].

## Discussion

The loss of function of *TMIE* is responsible for autosomal recessive non-syndromic HL (DFNB6). Previous studies have indicated the role of TMIE in the maintenance, maturation, and development of the sensory hair cells in the inner ear (reviewed in [20]). TMIE is considered one of the components of the mechanotransduction machinery on the surface of the stereocilia of hair cells [45, 46]. The mechanotransduction complex converts the mechanical stimuli of sound, gravity, and head accelerations into electrical signals. The transmission of these signals into the central nervous system is essential to the perception of sound [47].

Here, we reported three unrelated non-syndromic HL families due to pathogenic mutations in *TMIE* (Fig. 1). Initially, according to the ACMG guidelines for screening for genes related to HL [39], *GJB2* mutation testing was performed; the results were normal for three probands [12, 48, 49]. In the next step, WES was performed on the probands of three families. One novel ~ 10-kb deletion in family 1, a frameshift duplication (c.122_125dup) in family 2, and a missense (c.250 C > T) in family 3 were detected. These variants co-segregated with HL in families (Table 2; Fig. 2).

A homozygous deletion 9283 bp in NC_000003.12: g. 46,694,176-46703459del was reported for the first time in this document (Figs. 2C and 3). This deletion removes exon 1 of the *TMIE* gene and its surrounding intronic region, where the cis-regulatory elements are located (Fig. 3A). Therefore, this deletion can prevent transcription initiation or initiation from cryptic sites. As described in our previous review of the *TMIE* gene, no significant deletions to this gene have been associated with HL [20]. However, the total deletion of the *TMIE* gene has been previously reported in spinner [17] and circling mice [50, 51], which serve as animal models of humans regarding DFNB6. Both models are caused by a spontaneous 40-kb deletion on chromosome 9, which is analogous to human chromosome 3p21. The most important manifestations observed in these mice include HL, typical head tossing, circling, and hyperactivity.

The homozygous c.122_125dup variant inserts a 4-bp (CGCC) in exon 2, which is predicted to result in a frameshift, substituting 72 incorrect amino acids followed by a premature stop codon [16]. In the wild type of the TMIE protein, proline 43 is located on the extracellular loop between two transmembrane domains. This mutation was reported for the first time in an Indian family with a consanguineous marriage and three affected children [16].

The homozygous c.250C > T substitution was identified in exon 3 of the *TMIE* gene. This variant causes an arginine (Arg (R)) substitution to tryptophan (Trp (W)) at codon 84, which is located in a highly conserved residue within the intracellular carboxy-terminus of the TMIE protein. The emerging evidence demonstrates the importance of the carboxyl terminus of TMIE for its proper functioning. This domain participates in TMIE binding to PIP2 (phosphatidylinositol 4,5-bisphosphate) and TMC1 (Transmembrane channel-like 1), and correct collaboration of these components is essential for the proper functioning of the mechanotransduction channel [45, 46, 52]. The first report on p.R84W involved a family with a consanguineous marriage from southern India [16, 53]. Sirmaci et al. reported frequencies of 10.3% and 2.4% in Southeastern Anatolia and Turkey, respectively, while a haplotype analysis indicated that the mutation was due to a ‘Founder Effect’ that first occurred approximately 1250 years ago [54].

Moreover, in agreement with the previous report, no inner ear abnormality was observed in the affected members of this study [54].

An effective treatment for hearing impairment is still based on hearing amplification and cochlear implantation [55], even though no commonly used procedure can restore normal hearing to patients. A better understanding of the molecular mechanisms that cause hearing impairment can provide new perspectives for treatment [56, 57]. Early studies on the genetic causes of HL led to the introduction of high heterogeneity of single nucleotide variants as a responsible factor in this field. Later, the role of copy number variations (CNVs) as a common reason for HL was indicated using comprehensive genetic testing platforms [58, 59]. So far, CNVs have been reported in 29 non-syndromic HL-related genes, with *STRC*, *OTOA*, and *GJB2/GJB6* being the most well-known genes with deafness-causing CNVs [59]. To the best of our knowledge, no CNVs have been reported in *TMIE*. The present study expands the mutations varieties in the *TMIE* gene by introducing the first ~ 10-kb deletion. A noteworthy limitation of this study is that we could not perform a functional analysis of this variant in vitro or in vivo. TMIE is considered a critical protein in the mechanotransduction complex, which participates in functional mechanotransduction channel [45, 46, 52] and hair-bundle morphogenesis [21]. Further experimentation is needed to better understand the underlying cellular pathway of TMIE in developing HL. Such research could improve the quality of clinical diagnoses, medical management, genetic counseling, and prevention strategies for patients and their relatives.

## Conclusion

We have elucidated the role of the *TMIE* gene as a genetic cause of HL in three Iranian patients. We have also indicated the usefulness of WES, along with multiplex PCR, for identifying a novel ~ 10-kb causative deletion in *TMIE*. This study expands the mutational spectrum of the *TMIE* gene and indicates the importance of studying the connection between large deletions in this gene in HL.

## Supplementary Information

**Additional file 1** A) Categories of Auditory Performance-II (CAP-II) criteria. B) Speech intelligibility rating (SIR) categories.

## Data Availability

The whole-exome sequencing datasets used during this manuscript are available from the corresponding author on reasonable request. The novel variant was submitted in ClinVar database (accession number: SCV002098951).

## Abbreviations

TMIE: Transmembrane inner ear
DFNB6: Autosomal recessive deafness-6
HL: Hearing loss
WES: Whole-exome sequencing
HGMD: Human gene mutation database
DVD: Deafness variation database
ACMG: American College of Medical Genetics
AMP: Association for molecular pathology
CREs: Cis-regulatory elements
Arg: Arginine
Trp: Tryptophan
PIP2: Phosphatidylinositol 4,5-bisphosphate
TMC1: Transmembrane channel-like 1
CNVs: Copy number variations

## References

[1] Asghari A, Farhadi M, Daneshi A, Khabazkhoob M, Mohazzab-Torabi S, Jalessi M (2017). The prevalence of hearing impairment by age and gender in a population-based study. Iran J Public Health.

[2] Sloan-Heggen CM, Bierer AO, Shearer AE, Kolbe DL, Nishimura CJ, Frees KL (2016). Comprehensive genetic testing in the clinical evaluation of 1119 patients with hearing loss. Hum Genet.

[3] Haile LM, Kamenov K, Briant PS, Orji AU, Steinmetz JD, Abdoli A (2021). Hearing loss prevalence and years lived with disability, 1990–2019: findings from the global burden of disease study 2019. Lancet (London, England).

[4] Wilson BS, Tucci DL (2021). Addressing the global burden of hearing loss. Lancet (London, England).

[5] Olusanya BO, Neumann KJ, Saunders JE (2014). The global burden of disabling hearing impairment: a call to action. Bull World Health Organ.

[6] Nordvik O, Laugen Heggdal PO, Brannstrom J, Vassbotn F, Aarstad AK, Aarstad HJ (2018). Generic quality of life in persons with hearing loss: a systematic literature review. BMC Ear Nose Throat Disord.

[7] Huddle MG, Goman AM, Kernizan FC, Foley DM, Price C, Frick KD (2017). The economic impact of adult hearing loss: a systematic review. JAMA Otolaryngol Head Neck Surg.

[8] Azaiez H, Booth KT, Ephraim SS, Crone B, Black-Ziegelbein EA, Marini RJ (2018). Genomic landscape and mutational signatures of deafness-associated genes. Am J Hum Genet.

[9] Falah M, Houshmand M, Mahmoudian S, Emamdjomeh H, Ghavami Y, Farhadi M (2012). The anticipation and inheritance pattern of c.487A>G mutation in the GJB2 gene. Arch Iran Med.

[10] Dowlati MA, Derakhshandeh-Peykar P, Houshmand M, Farhadi M, Shojaei A, Fallah M (2013). Novel nucleotide changes in mutational analysis of mitochondrial 12SrRNA gene in patients with nonsyndromic and aminoglycoside-induced hearing loss. Mol Biol Rep.

[11] Falah M, Houshmand M, Najafi M, Balali M, Mahmoudian S, Asghari A (2016). The potential role for use of mitochondrial DNA copy number as predictive biomarker in presbycusis. Ther Clin Risk Manag.

[12] Falah M, Houshmand M, Balali M, Asghari A, Bagher Z, Alizadeh R (2020). Role of GJB2 and GJB6 in Iranian nonsyndromic hearing impairment: from molecular analysis to literature reviews. Fetal Pediatr Pathol.

[13] Shearer AE, Hildebrand MS, Smith RJ. Hereditary hearing loss and deafness overview. J GeneReviews® 2017.

[14] Shearer AE, Smith RJ (2015). Massively parallel sequencing for genetic diagnosis of hearing loss: the new standard of care. Otolaryngol Head Neck Surg.

[15] Vafaee-Shahi M, Farhadi M, Razmara E, Morovvati S, Ghasemi S, Abedini SS (2021). Novel phenotype and genotype spectrum of NARS2 and literature review of previous mutations. Ir J Med Sci.

[16] Naz S, Giguere CM, Kohrman DC, Mitchem KL, Riazuddin S, Morell RJ (2002). Mutations in a novel gene, TMIE, are associated with hearing loss linked to the DFNB6 locus. Am J Hum Genet.

[17] Mitchem KL, Hibbard E, Beyer LA, Bosom K, Dootz GA, Dolan DF (2002). Mutation of the novel gene Tmie results in sensory cell defects in the inner ear of spinner, a mouse model of human hearing loss DFNB6. Hum Mol Genet.

[18] Gleason MR, Nagiel A, Jamet S, Vologodskaia M, Lopez-Schier H, Hudspeth AJ (2009). The transmembrane inner ear (Tmie) protein is essential for normal hearing and balance in the zebrafish. Proc Natl Acad Sci USA.

[19] Chung WH, Kim KR, Cho YS, Cho DY, Woo JH, Ryoo ZY (2007). Cochlear pathology of the circling mouse: a new mouse model of DFNB6. Acta Otolaryngol.

[20] Farhadi M, Razmara E, Balali M, Hajabbas Farshchi Y, Falah M (2021). How Transmembrane Inner Ear (TMIE) plays role in the auditory system: a mystery to us. J Cell Mol Med.

[21] Krey JF, Chatterjee P, Dumont RA, O'Sullivan M, Choi D, Bird JE (2020). Mechanotransduction-dependent control of stereocilia dimensions and row identity in inner hair cells. Curr Biol.

[22] Alford RL, Arnos KS, Fox M, Lin JW, Palmer CG, Pandya A (2014). American college of medical genetics and genomics guideline for the clinical evaluation and etiologic diagnosis of hearing loss. Genet Med.

[23] Falah M, Farhadi M, Kamrava SK, Mahmoudian S, Daneshi A, Balali M (2017). Association of genetic variations in the mitochondrial DNA control region with presbycusis. Clin Interv Aging.

[24] Karczewski KJ, Francioli LC, Tiao G, Cummings BB, Alfoldi J, Wang Q (2020). The mutational constraint spectrum quantified from variation in 141,456 humans. Nature.

[25] Fairley S, Lowy-Gallego E, Perry E, Flicek P (2020). The International Genome sample resource (IGSR) collection of open human genomic variation resources. Nucleic Acids Res.

[26] Smigielski EM, Sirotkin K, Ward M, Sherry ST (2000). dbSNP: a database of single nucleotide polymorphisms. Nucleic Acids Res.

[27] Fattahi Z, Beheshtian M, Mohseni M, Poustchi H, Sellars E, Nezhadi SH (2019). Iranome: a catalog of genomic variations in the Iranian population. Hum Mutat.

[28] Schwarz JM, Cooper DN, Schuelke M, Seelow D (2014). MutationTaster2: mutation prediction for the deep-sequencing age. Nat Methods.

[29] Adzhubei IA, Schmidt S, Peshkin L, Ramensky VE, Gerasimova A, Bork P (2010). A method and server for predicting damaging missense mutations. Nat Methods.

[30] Sim NL, Kumar P, Hu J, Henikoff S, Schneider G, Ng PC (2012). SIFT web server: predicting effects of amino acid substitutions on proteins. Nucleic Acids Res.

[31] Choi Y, Chan AP (2015). PROVEAN web server: a tool to predict the functional effect of amino acid substitutions and indels. Bioinformatics.

[32] Rentzsch P, Witten D, Cooper GM, Shendure J, Kircher M (2019). CADD: predicting the deleteriousness of variants throughout the human genome. Nucleic Acids Res.

[33] Desmet FO, Hamroun D, Lalande M, Collod-Beroud G, Claustres M, Beroud C (2009). Human splicing finder: an online bioinformatics tool to predict splicing signals. Nucleic Acids Res.

[34] Spradling AC, Stern D, Beaton A, Rhem EJ, Laverty T, Mozden N (1999). The berkeley drosophila genome project gene disruption project: single P-element insertions mutating 25% of vital drosophila genes. Genetics.

[35] Brunak S, Engelbrecht J, Knudsen S (1991). Prediction of human mRNA donor and acceptor sites from the DNA sequence. J Mol Biol.

[36] Stenson PD, Ball EV, Mort M, Phillips AD, Shaw K, Cooper DN (2012). The Human gene mutation database (HGMD) and its exploitation in the fields of personalized genomics and molecular evolution. Curr Protoc Bioinform.

[37] Landrum MJ, Chitipiralla S, Brown GR, Chen C, Gu B, Hart J (2020). ClinVar: improvements to accessing data. Nucleic Acids Res.

[38] Kohler S, Vasilevsky NA, Engelstad M, Foster E, McMurry J, Ayme S (2017). The human phenotype ontology in 2017. Nucleic Acids Res.

[39] Oza AM, DiStefano MT, Hemphill SE, Cushman BJ, Grant AR, Siegert RK (2018). Expert specification of the ACMG/AMP variant interpretation guidelines for genetic hearing loss. Hum Mutat.

[40] Rozen S, Skaletsky H. Primer3 on the WWW for general users and for biologist programmers. In: *Bioinformatics methods and protocols.* Springer; 2000: 365–386. 10.1385/1-59259-192-2:365 10547847

[41] Allen MC, Nikolopoulos TP, O'Donoghue GM (1998). Speech intelligibility in children after cochlear implantation. Am J Otol.

[42] Gilmour L: The inter-rater reliability of categories of auditory performance-II (CAP)-II. University of Southampton; 2010.

[43] MacDonald JR, Ziman R, Yuen RK, Feuk L, Scherer SW (2014). The Database of genomic variants: a curated collection of structural variation in the human genome. Nucleic Acids Res.

[44] Kent WJ, Sugnet CW, Furey TS, Roskin KM, Pringle TH, Zahler AM (2002). The human genome browser at UCSC. Genome Res.

[45] Cunningham CL, Qiu X, Wu Z, Zhao B, Peng G, Kim YH (2020). TMIE Defines pore and gating properties of the mechanotransduction channel of mammalian cochlear hair cells. Neuron.

[46] Pacentine IV, Nicolson T (2019). Subunits of the mechano-electrical transduction channel, Tmc1/2b, require Tmie to localize in zebrafish sensory hair cells. PLoS Genet.

[47] Gillespie PG, Muller U (2009). Mechanotransduction by hair cells: models, molecules, and mechanisms. Cell.

[48] Falah M, Houshmand M, Akbaroghli S, Mahmodian S, Ghavami Y, Farhadi M (2011). Profile of Iranian GJB2 mutations in young population with novel mutation. Iran J Basic Med Sci.

[49] Daneshi A, Hassanzadeh S, Emamdjomeh H, Mohammadi SH, Arzhangi S, Farhadi M (2011). Prevalence of GJB2-associated deafness and outcomes of cochlear implantation in Iran. J Laryngol Otol.

[50] Cho KI, Lee JW, Kim KS, Lee EJ, Suh JG, Lee HJ (2003). Fine mapping of the circling (cir) gene on the distal portion of mouse chromosome 9. Comp Med.

[51] In Cho K, Suh J-G, Woong Lee J, Hwa Hong S, Kang T-C, Oh Y-S (2006). The circling mouse (C57BL/6J-cir) has a 40-kilobase genomic deletion that includes the transmembrane inner ear (tmie) gene. Comp Med.

[52] Zhao B, Wu ZZ, Grillet N, Yan LX, Xiong W, Harkins-Perry S (2014). TMIE Is an essential component of the mechanotransduction machinery of cochlear hair cells. Neuron.

[53] Fukushima K, Ramesh A, Srisailapathy CR, Ni L, Wayne S, O'Neill ME (1995). An autosomal recessive nonsyndromic form of sensorineural hearing loss maps to 3p-DFNB6. Genome Res.

[54] Sırmacı A, Öztürkmen-Akay H, Erbek S, Incesulu A, Duman D, Taşır-Yılmaz S (2009). A founder TMIE mutation is a frequent cause of hearing loss in southeastern Anatolia. J Clin Genet.

[55] Daneshi A, Ajalloueyan M, Ghasemi MM, Hashemi BS, Emamjome H, Farhadi M (2015). Complications in a series of 4400 paediatric cochlear implantation. Int J Pediatr Otorhinolaryngol.

[56] Delmaghani S, El-Amraoui A (2020). Inner ear gene therapies take off: current promises and future challenges. J Clin Med.

[57] Ding N, Lee S, Lieber-Kotz M, Yang J, Gao X (2021). Advances in genome editing for genetic hearing loss. Adv Drug Deliv Rev.

[58] Shearer AE, Kolbe DL, Azaiez H, Sloan CM, Frees KL, Weaver AE (2014). Copy number variants are a common cause of non-syndromic hearing loss. Genome Med.

[59] Abbasi W, French CE, Rockowitz S, Kenna MA, Eliot SA (2021). Evaluation of copy number variants for genetic hearing loss: a review of current approaches and recent findings. Hum Genet.

