# Supplementary material for: Analysis of TMIE gene mutations including the first large deletion of exon 1 with autosomal recessive non-syndromic deafness
Source: BMC Med Genomics. 2022 Jun 16;15:133. doi: 10.1186/s12920-022-01287-9 (PMC9204965; doi:10.1186/s12920-022-01287-9)
Supplement: Supplementary file 1 — Additional file 1 A) Categories of Auditory Performance-II (CAP-II) criteria. B) Speech intelligibility rating (SIR) categories. [file 12920_2022_1287_MOESM1_ESM.docx]

**Supplementary File 1.** **A)** Categories of Auditory Performance-II (CAP-II) criteria. **B)** Speech intelligibility rating (SIR) categories.

**A)**

| Category | Criteria |
| --- | --- |
| 9 | Use of phone with unknown speaker in unpredictable context. |
| 8 | Follows group conversation in a reverberant room or where there is some interfering noise, such as a classroom or restaurant. |
| 7 | Use of telephone with known listener. |
| 6 | Understanding of conversation without lip-reading. |
| 5 | Understanding of common phrases without lip-reading. |
| 4 | Discrimination of some speech sounds without lip-reading. |
| 3 | Identification of environmental sounds. |
| 2 | Response to speech sounds (e.g. “go”). |
| 1 | Awareness of environmental sounds. |
| 0 | No awareness of environmental sounds. |

**B)**

| Category | Category |
| --- | --- |
| 5 | Connected speech is intelligible to all listeners. Child is understood easily in everyday contexts |
| 4 | Connected speech is intelligible to a listener who has a little experience of a deaf person's speech |
| 3 | Connected speech is intelligible to a listener who concentrates and lipreads |
| 2 | Connected speech is unintelligible. Intelligible speech is developing in single words when context and lip-reading cues are available |
| 1 | Connected speech is unintelligible. Pre-recognizable words in spoken language. Primary mode of communication may be manual |
